# Supplementary material for: The Clinical Impact of the Omicron Variant on Octogenarian Hospitalized COVID-19 Patients: The Results from CoviCamp Cohort
Source: Biomedicines. 2025 Jun 26;13(7):1563. doi: 10.3390/biomedicines13071563 (PMC12292787; doi:10.3390/biomedicines13071563)
Supplement: Supplementary file 1 [file biomedicines-13-01563-s001.zip › biomedicines-3635461-supplementary.pdf]

SUPPLEMENTARY DATA

Supplementary Table S1: STROBE recommendations for an observational study

	Item No.	Recommendation	Page No.
**Title and abstract**	1	(*a*) Indicate the study's design with a commonly used term in the title or the abstract	1–2
		(*b*) Provide in the abstract an informative and balanced summary of what was done and what was found	2
**Introduction**			
Background/rationale	2	Explain the scientific background and rationale for the investigation being reported	4
Objectives	3	State specific objectives, including any prespecified hypotheses	4
**Methods**			
Study design	4	Present key elements of study design early in the paper	5
Setting	5	Describe the setting, locations, and relevant dates, including periods of recruitment, exposure, follow-up, and data collection	5
Participants	6	(*a*) *Cohort study*—Give the eligibility criteria, and the sources and methods of selection of participants. Describe methods of follow-up	5
Variables	7	Clearly define all outcomes, exposures, predictors, potential confounders, and effect modifiers. Give diagnostic criteria, if applicable	6
Data sources/ measurement	8	For each variable of interest, give sources of data and details of methods of assessment (measurement). Describe comparability of assessment methods if there is more than one group	6
Bias	9	Describe any efforts to address potential sources of bias	6
Study size	10	Explain how the study size was arrived at	7
Quantitative variables	11	Explain how quantitative variables were handled in the analyses. If applicable, describe which groupings were chosen and why	6
Statistical methods	12	(*a*) Describe all statistical methods, including those used to control for confounding	6
		(*b*) Describe any methods used to examine subgroups and interactions	6

	Item No.	Recommendation	Page No.
		(*c*) Explain how missing data were addressed	6
		(*d*) *Cohort study*—If applicable, explain how loss to follow-up was addressed	5
		(*e*) Describe any sensitivity analyses	6
**Results**			
Participants	13	(a) Report numbers of individuals at each stage of study—e.g., numbers potentially eligible, examined for eligibility, confirmed eligible, included in the study, completing follow-up, and analysed	7
		(b) Give reasons for non-participation at each stage	Suppl. Figure 1
		(c) Consider use of a flow diagram	Suppl Figure 1
Descriptive data	14	(a) Give characteristics of study participants (e.g., demographic, clinical, social) and information on exposures and potential confounders	7
		(b) Indicate number of participants with missing data for each variable of interest	7
		(c) *Cohort study*—Summarise follow-up time (e.g., average and total amount)	7
		Cohort study—Report numbers of outcome events or summary measures over time	*7*
Main results	16	(*a*) Give unadjusted estimates and, if applicable, confounder-adjusted estimates and their precision (eg, 95% confidence interval). Make clear which confounders were adjusted for and why they were included	7
		(*b*) Report category boundaries when continuous variables were categorized	7
		(*c*) If relevant, consider translating estimates of relative risk into absolute risk for a meaningful time period	NA
Other analyses	17	Report other analyses done—e.g., analyses of subgroups and interactions, and sensitivity analyses	7
**Discussion**			
Key results	18	Summarise key results with reference to study objectives	8–9

	Item No.	Recommendation	Page No.
Limitations	19	Discuss limitations of the study, taking into account sources of potential bias or imprecision. Discuss both direction and magnitude of any potential bias	10
Interpretation	20	Give a cautious overall interpretation of results considering objectives, limitations, multiplicity of analyses, results from similar studies, and other relevant evidence	9–10
Generalisability	21	Discuss the generalisability (external validity) of the study results	9–10
**Other information**			
Funding	22	Give the source of funding and the role of the funders for the present study and, if applicable, for the original study on which the present article is based	11

Supplementary Table S2: Demografic, clinical and laboratory characteristic of patients included grouped by age.

	**65–79**	>80	***p* Value**
**Number of patients**, *n (%)*	652	341	-
**Age**, *median [IQR]*	71 [68–75]	84 [82–87]	**0.0001**
**Males**, *n (%)*	423 (64.9)	161 (47.2)	**0.0001**
**Omicron variant,** *n° (%)*	0(0)	79 (23.2)	**0.0001**
**Charlson Comorbidity Index,** *median [IQR]*	3 (1–5)	3 (1–5)	**0.028**
**Hypertension**, *n (%)*	295 (46.2)	170 (51.7)	0.109
**Diabetes**, *n (%)*	135 (21)	82 (24.8)	0.172
**Overweight/Obesity**, *n (%)*	53 (12.5)	17 (9.3)	0.261
**COPD**, *n (%)*	66 (10.3)	59 (17.9)	**0.001**
**Cardiovascular pathology**, *n (%)*	180 (28)	102 (30.9)	0.371
**Dementia**, *n (%)*	28 (5.2)	24 (8.7)	**0.048**
**Fever**, *n (%)*	359 (61.1)	146 (49.8)	**0.002**
**HIV**, *n (%)*	4 (0.7)	2 (0.6)	1
**Active tumor**, *n (%)*	41 (6.4)	46 (13.9)	**0.0001**
**Chronic liver disease**, *n (%)*	24 (3.8)	16 (4.9)	0.428
**CKD**, *n (%)*	62 (9.7)	37 (11.3)	0.448
**Smoking**, *n (%)*	23 (4.7)	30 (12.7)	**0.0001**
**Cough,** *n (%)*	213 (36.5)	76 (26.2)	**0.002**
**Asthenia**, *n (%)*	158 (29.1)	56 (20.4)	**0.007**
Ageusia/Dysgeusia, *n (%)*	31 (5.4)	7 (2.3)	**0.037**
Anosmia/hyposmia , *n (%)*	23 (4)	7 (2.6)	0.298
Diarrhea, *n (%)*	26 (4.5)	14 (4.8)	0.840
**Skin lesions**, *n (%)*	2 (0.4)	7 (2.5)	**0.008**
**Dyspnea**, *n (%)*	387(66)	182 (62.8)	0.338
**WBC**, *median [IQR]*	7700 [5410–10540]	7430 [5280–9730]	0.154
**Lymphocytes**, *median [IQR]*	814 [580–1200]	940 [643–1370]	**0.007**
**Neu**, *median [IQR]*	6330 [4053–8848]	5797 [3641–8150]	**0.046**
**INR**, *median [IQR]*	1.09 [1.02–1.19]	1.10 [1.03–1.21]	0.133
**LDH**, *median [IQR]*	305 [233–413]	280 [215–385]	**0.007**
**Creatinine**, *median [IQR]*	0.88 [0.7–1.13]	0.89 [0.71–1.10]	0.566
**CPK**, *median [IQR]*	86.5 [47–189.5]	65 [36–118]	**0.0001**
**GOT**, *median [IQR]*	32 [21–47]	27 [20–41]	**0.005**

**GPT**, *median [IQR]*	32 [20–54]	27 [19–48]	**0.048**
**Total bilirubinemia**, *median [IQR]*	0.590 [0.420–0.820]	0.620 [0.43–0.87]	0.228
**Direct bilirubinemia,** *median [IQR]*	0.21 [0.16–0.31]	0.23 [0.18–0.375]	**0.049**
**P/F,** *median [IQR]*	233 [152–323]	238.5 [145–314]	0.608
**Time of negativization,** *median [IQR]*	14 [3–23]	14 [0–20]	0.161
**Hospitalization days**, *median [IQR]*	15 [10–22]	14 [8–19]	**0.005**
**Severe/critical outcome,** *n (%)*	193 (34.2)	86 (30.3)	0.249
**Death,** *n (%)*	72 (12.8)	31 (10.9)	0.436

Supplementary Table S3: Analysis of covariates associated with all causes in-hospital mortality.

Variables in the Equation

	B	S.E.	Wald	df	Sig.	Exp(B)
COPD	0.181	0.754	0.058	1	0.810	1.198
Neoplasia	2.006	0.721	7.737	1	**0.005**	7.435
Cardiovascular Disease	1.114	0.752	2.192	1	0.139	3.047
Omicron Era	−2.269	1.573	2.080	1	00.149	0.103
Age	0.168	0.137	1.499	1	0.221	1.183
CKD	1.249	0.820	2.318	1	0.128	3.485
Type II Diabetes Mellitus	0.580	0.697	0.693	1	0.405	1.787
Obesity	0.452	0.932	0.236	1	0.627	1.572
Constant	−17.680	11.576	2.333	1	0.127	0.000
